# Dose–response association of handgrip strength and risk of depression: a longitudinal study of 115 601 older adults from 24 countries

**DOI:** 10.1192/bjp.2022.178

**Published:** 2023-03

**Authors:** Rubén López-Bueno, Joaquín Calatayud, Lars Louis Andersen, José Casaña, Ai Koyanagi, Borja del Pozo Cruz, Lee Smith

**Affiliations:** Department of Physical Medicine and Nursing, University of Zaragoza, Spain; Exercise Intervention for Health Research Group (EXINH-RG), Department of Physiotherapy, University of Valencia, Spain; National Research Centre for the Working Environment, Denmark; Research and Development Unit, Parc Sanitari Sant Joan de Déu, CIBERSAM, ICREA, Spain; Centre for Active and Healthy Ageing, Department of Sports Science and Clinical Biomechanics, University of Southern Denmark, Denmark; Centre for Health, Performance, and Wellbeing, Anglia Ruskin University, UK

**Keywords:** Muscle, mental disorder, epidemiology, prevention, burden of disease

## Abstract

**Background:**

Prior research has solely focused on the association between handgrip strength and risk of depression in single countries or general populations, but more knowledge is required from wider-spread cohorts and target populations.

**Aims:**

This study aimed to investigate the association between handgrip strength and risk of depression using repeated measures in adults aged 50 years and over.

**Method:**

Data on handgrip strength and risk of depression were retrieved from the Survey of Health, Ageing and Retirement in Europe (SHARE) waves 1, 2, 4, 5, 6 and 7, using a hand dynamometer (Smedley, S Dynamometer, TTM) and the EURO-D 12-item scale, respectively. Time-varying exposure and covariates were modelled using both Cox regression and restricted cubic splines.

**Results:**

A total of 115 601 participants (mean age 64.3 years (s.d. = 9.9), 54.3% women) were followed-up for a median of 7.3 years (interquartile range: 3.9–11.8) and 792 459 person-years. During this period, 30 208 (26.1%) participants experienced a risk of depression. When modelled as a continuous variable, we observed an inverse significant association for each kg increase of handgrip strength and depression up to 40 kg in men and up to 27 kg in women.

**Conclusions:**

Being physically strong may serve as a preventive factor for depression in older adults, but this is limited up to a maximum specific threshold for men and women.

## Background

Depression is considered a contemporary chronic condition that can impair normal mental and physical functioning; the manifestation of depression varies among individuals, but often involves lack of energy, low mood, sadness, insomnia and an inability to enjoy life.^1^ The incidence of depression disorders increased by 50% over the period from 1990 to 2017 worldwide, and the COVID-19 pandemic has increased the prevalence of depression compared with pre-pandemic levels.^2,3^

Importantly, depression has been observed to increase the risk of all-cause and cardiovascular mortality in middle-aged and older adults.^4,5^ It has also been associated with significantly increased risks for hypertension, myocardial infarction, stroke, physical impairment and suicidal attempts, and is one of the leading causes of global disease burden in terms of disability-adjusted life-years, years lived with disability and years of life lost.^6,7^ Thus, because depression represents a major public health concern, studies aiming at examining preventive factors to tackle the increase in depression are required. In fact, early prevention is estimated to reduce 20% to 25% incident depression in high-income countries, which warrants the implementation of preventive measures.^8^

In this regard, there is a growing body of research examining the association between muscle strength, using handgrip as an estimator, and depression in healthy middle-aged and older adults.^9,10^ Handgrip strength is an easy-to-use, fast and reliable indicator of both sarcopenia (age-related loss of muscle mass) and dynapenia (age-related loss of muscle strength). As both have been associated with depression, the plausibility of a regulatory role of skeletal muscle on brain function affecting this condition exists.^11–14^ Interestingly, exercise also seems to play a key role in the aforementioned relationships, as it can improve muscle strength and muscle mass, downregulates systemic inflammation and improves neuroplasticity, neuroendocrine and oxidative stress responses.^15–17^ Furthermore, handgrip strength has also been observed as a more useful single marker of frailty (a clinical syndrome in older adults characterised by an increased risk for poor health outcomes such as falls, disability, hospital admissions and mortality) for older people of similar age than using chronological age alone.^18^

## Aims

Nevertheless, study designs of previous evidence rely on either cross-sectional or prospective cohort studies (i.e. one-single base level of handgrip strength) mostly focused on a specific country, and not accounting for time-varying changes of both handgrip strength and relevant covariates.^10,19–21^ Furthermore, there is mixed evidence on the extent to which handgrip strength levels may associate with lower risk of depression, with study results ranging from weak to strong associations.^9,21,22^ Thus, higher-quality research with representative samples from different countries is required to better clarify the strength of such an association and to confirm directionality. Therefore, to circumvent limitations from prior research, we aimed to investigate the association between levels of handgrip strength and risk of depression among a large and representative sample of adults from 24 countries using repeated measures.

## Method

### Study design and population

The present study included data from waves 1, 2, 4, 5, 6 and 7 of the Survey of Health, Ageing and Retirement in Europe (SHARE), a survey recruiting individuals aged 50 years or older residing in European countries and Israel using a panel methodology.^23,24^ Wave 3 was discarded because it did not include data on handgrip strength.

**Fig. 1:**
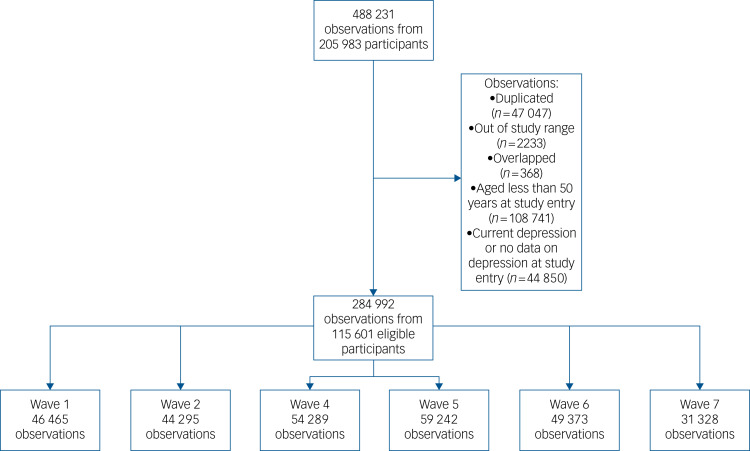
Study profile.

SHARE uses a multistage stratified sampling design in which involved countries are divided into different strata in relation to geographical area, and municipalities or zip codes within these strata served as primary sampling units.^25^ Data collection for each SHARE wave was carried out biannually through home computer-assisted personal interviews from February 2004 to January 2019. SHARE uses ex-ante harmonised interviews, and new respondents were added in each wave to compensate for losses.^25^ Only participants aged 50 years and over at study entry with no depression at study entry were included in the present study (*n* = 115 601). Figure 1 shows more descriptive details of the study sample.

The present study was reported according to Strengthening the Reporting of Observational Studies in Epidemiology (STROBE).^26^ The authors assert that all procedures contributing to this work comply with the ethical standards of the relevant national and institutional committees on human experimentation and with the Helsinki Declaration of 1975, as revised in 2008. All procedures involving human participants were approved by the Ethics Committee of Research in Humans of the University of Valencia (registered code 1510464). Written informed consent was obtained from all subjects.

### Handgrip strength (exposure)

Using a handheld dynamometer (Smedley, S Dynamometer, TTM, Tokio, 100 kg), each hand was measured two times by trained interviewers. Participants were instructed to set their elbow in a 90° angle flexion while either standing or sitting, with a neutral wrist position, and upper arm set vertically against the trunk. Trained interviewers provided standardised instructions to squeeze the dynamometer with maximum effort for 2 s. Handgrip strength was considered as the maximum value of either hand. To account for gender differences and meaningful results, handgrip strength was categorised into gender-stratified tertiles. Additional analyses also accounted for handgrip strength as a continuous variable.

### Risk of depression (outcome)

Participants were followed throughout the study period to determine whether they were at risk of depression using the EURO-D 12-item scale. Both description and validation of the scale have been described previously.^27^ For the purpose of this study, we used an optimal cut-off point of ≥4 depression symptoms, which has been identified as a clinically significant case of depression.^27,28^

### Covariates

Based on a literature review on the topic,^29,30^ we explored potential causal and confounding pathways between handgrip strength and risk of depression using a directed acyclic graph (Supplementary Figure 1; available online at https://doi.org/10.1192/bjp.2022.178). Self-reported gender, age, education, country, body mass index, physical inactivity, smoking, alcohol consumption, status regarding whether living with a partner, wave of inclusion, chronic diseases, prescribed drugs consumption, and fruits and vegetables consumption were identified as potential confounders.

- Education was self-reported by participants and thereafter coded using the 1997 version of the International Standard Classification of Education.
- Body mass index was calculated from self-reported height and weight and grouped into four categories according to standards proposed by the World Health Organization.
- Physical inactivity was assessed through two questions: ‘How often do you engage in vigorous physical activity such as sports, heavy housework, or a job that involves physical labour’, and ‘How often do you engage in activities that require a moderate level of energy such as gardening, cleaning the car or going for a walk?’. Participants selecting the option of ‘Hardly ever, or never’ to the two questions were considered physically inactive.
- Smoking habits were assessed through the question ‘Have you ever smoked cigarettes, cigars, cigarillos or a pipe daily for a period of at least 1 year?’, whereas alcohol consumption was estimated through the following question: ‘How many days a week did you consume alcohol during the last 6 months?’, and answers comprised the following possible options: ‘Almost every day’, ‘Five or six days a week’, ‘Three or four days a week’, ‘Once or twice a week’, ‘Once or twice a month’, ‘Less than once a month’, ‘Not at all in the last 6 months’, ‘Refusal to answer’, or ‘Don't know’.
- Living with a partner was estimated through asking if the surveyed individual was living with any partner/spouse and responses included ‘yes’, ‘no, or ‘refused’ as options.
- The number of chronic diseases was provided by the surveyed individuals from a list comprising 14 common chronic diseases.
- Drug consumption (i.e. medicines for treating chronic conditions) was assessed through the following question: ‘Do you currently use drugs at least once a week for problems mentioned on this card?’ This variable was re-coded into the categories ‘None’ for those who answered such option in the survey, and ‘Any’ for those who took one or more of a list of drugs.
- Fruits and vegetables consumption were measured using a scale of 1 (less than once a week) to 5 (every day).

Details on these covariates are provided elsewhere.^25^

### Statistical analyses

We conducted all statistical analyses with Stata version 16.1 (StataCorp, Texas, USA). We used Cox regression to estimate the hazard ratios (HRs) for first experienced risk of depression within the examined period using repeated measures of handgrip strength. Months from study entry were set as the timescale and the follow-up continued until either a first depression onset or the end of follow-up occurred. Two models were tested: a model with both gender and age at the time of the interview as time-invariant confounders (model A) and a fully adjusted model (model B) including model A confounders along with other time-invariant confounders (age at the time of interview, gender, country, wave of inclusion, and education) and time-variant confounders (body mass index, physical inactivity, smoking, alcohol consumption, living with a partner, wave, chronic diseases, prescribed drugs consumption and fruits and vegetables consumption).

Imputations for missing values (24.7%) were calculated using multiple imputation including the outcome as well as all the covariates without missing values in the equation. After assessing interactions between handgrip strength and all the covariates, no significant interaction was detected. All the analyses were weighted according to each country population (Supplementary Table 1). We provided the results using random forest plots. Additionally, we assessed the dose–response associations of handgrip strength (modelled as a continuous exposure) with depression using restricted cubic splines to allow for potential non-linearity; we trimmed observations less than 5% and greater than 95% of the distribution and pre-specified knots placed at the 5th, 25th, 50th, 75th and 95th percentiles of the exposure distribution.

Departure from linearity was checked with a Wald test assessing the null hypothesis that the coefficient of the fifth spline was equal to zero. We assumed linearity for values below the 5th percentile and for values above the 95th percentile. Multiple imputation of missing values was conducted using a chained equation including all the covariates and the outcome variable. We assumed that data were missing at random. Overall, we imputed five data-sets using the Stata native command (mi impute). No auxiliary variables were used for this purpose.

Results of the study are reported as HRs with 95% CIs and levels of significance were set at *P* < 0.05.

### Sensitivity analyses

To check the robustness of the estimates, we conducted complete-case analyses (Supplementary Figure 2). Moreover, to minimise the potential influence of reverse causality, we conducted analyses excluding participants who experienced depression within 2 years of follow-up (Supplementary Figure 3). Finally, we additionally accounted for mortality and attrition as competing risk in our estimations through Fine–Gray models (Supplementary Figure 4). We also accounted for the Nelson–Aalen cumulative hazard estimate for the survival time in the imputation model (Supplementary Figure 5).^31^

## Results

### Demographics

The final sample included 115 601 participants with a mean age of 64.3 (s.d. = 9.9) years at study entry (Table 1) of which 54.3% were women. During a median of 7.3 years of follow-up (interquartile range, 3.9–11.8) and 792 459 person-years, 30 208 (26.1%) participants experienced a risk of depression.

**Table 1:** Characteristics of participants at study entry (*n* = 115 601)^a^

	Value
Age, years, mean (s.d.)	64.3 (9.9)
Gender, *n* (%)
Men	52 858 (45.7)
Women	62 743 (54.3)
Body mass index, kg/m^2^, *n* (%)
Underweight (<18.5 kg/m^2^)	1239 (1.1)
Normal (18.5–<25 kg/m^2^)	40 602 (35.1)
Overweight (25–<30 kg/m^2^)	47 683 (41.3)
Obese (≥30 kg/m^2^)	23 396 (20.2)
Missing	2681 (2.3)
Education,^b^ *n* (%)
None	4781 (4.1)
Primary	21 374 (18.5)
Lower secondary	20 889 (18.1)
Upper secondary	38 699 (33.5)
Post-secondary non-tertiary	4905 (4.2)
First stage of tertiary	23 437 (20.3)
Second stage of tertiary	839 (0.7)
Other	475 (0.4)
Missing	202 (0.2)
Current smoking habit, *n* (%)
No	65 913 (57.0)
Yes	31 890 (27.6)
Missing	17 798 (15.4)
Alcohol consumption, *n* (%)
Almost every day	18429 (15.9)
Five or six days a week	2973 (2.6)
Three or four days a week	7423 (6.4)
Once or twice a week	20 771 (18.0)
Once or twice a month	14 492 (12.5)
Less than once a month	10 722 (9.3)
Not at all in the last 6 months	36 369 (31.5)
Missing	4422 (3.8)
Living with a partner/spouse, *n* (%)
No	28 171 (24.4)
Yes	87 430 (75.6)
Number of chronic diseases, mean (s.d.)	1.6 (1.5)
Missing	143 (0.1)
Prescribed drug consumption, *n* (%)
No	81 140 (70.2)
Yes	34 294 (29.7)
Missing	167 (0.1)
Fruits and vegetables consumption, *n* (%)
Every day	64 529 (55.8)
3–6 times a week	15756 (13.6)
Twice a week	3625 (3.1)
Once a week	1239 (1.1)
Less than once a week	820 (0.7)
Missing	29 632 (25.6)
Physical inactivity, *n* (%)
No	92 937 (80.4)
Yes	10 009 (8.7)
Missing	12 655 (11.0)
Country, *n* (%)
Austria	5947 (5.1)
Belgium	8811 (7.6)
Bulgaria	1944 (1.7)
Croatia	2562 (2.2)
Czech Republic	7676 (6.6)
Denmark	5421 (4.7)
Estonia	7158 (6.2)
Finland	1975 (1.7)
France	7238 (6.3)
Germany	7912 (6.8)
Greece	5550 (4.8)
Hungary	2417 (2.1)
Ireland	825 (0.7)
Israel	3412 (3.0)
Italy	7525 (6.5)
Luxembourg	1897 (1.6)
Netherlands	5618 (4.9)
Poland	4261 (3.7)
Portugal	1901 (1.6)
Romania	2055 (1.8)
Slovenia	5089 (4.4)
Spain	7937 (6.9)
Switzerland	4307 (3.7)
Sweden	6163 (5.3)
Handgrip strength (kg), mean (s.d.)	34.3 (12.1)
Handgrip strength (kg), *n* (%)	
Third 1	39 787 (34.4)
Third 2	39 079 (33.8)
Third 3	34 602 (29.9)
Missing	2133 (1.9)

a. Missing values were imputed.

b. Based on International Standard Classification of Education (ISCED) 1997.

**Fig. 2:**
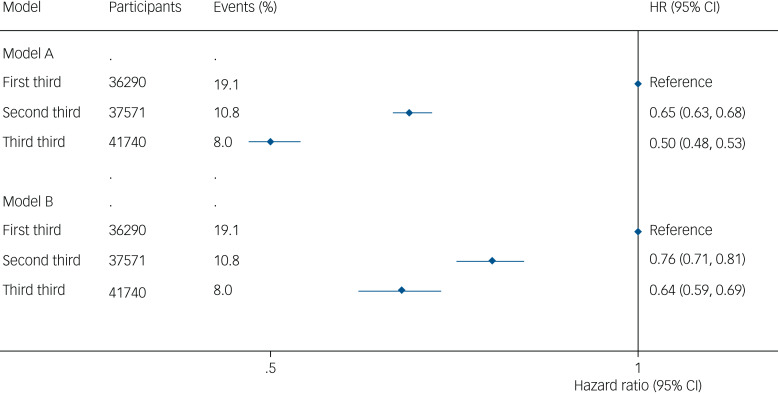
Prospective associations between handgrip strength (kg) and risk of depression model A, adjusted for age and gender. Model B, adjusted for age, gender, education, country, body mass index, wave, physical inactivity, smoking, alcohol, partner, chronic diseases, prescribed drugs consumption and fruits and vegetables consumption. HR, hazard ratio.

Results from the model adjusted for gender and age only (model A) showed that handgrip strength significantly reduced the risk of depression among participants in the second-third (HR 0.65, 95% CI 0.63–0.68) and the final third (HR 0.50, 95% CI 0.48–0.53) (reference first-third) (Figure 2).

The observed associations were consistent in the fully adjusted model (model B), which slightly attenuated the risk of depression in the second-third (HR 0.76, 95% CI, 0.71–0.81) and the final third (HR 0.64; 95% CI 0.59–0.69) (reference: first-third) (Figure 2).

**Fig. 3:**
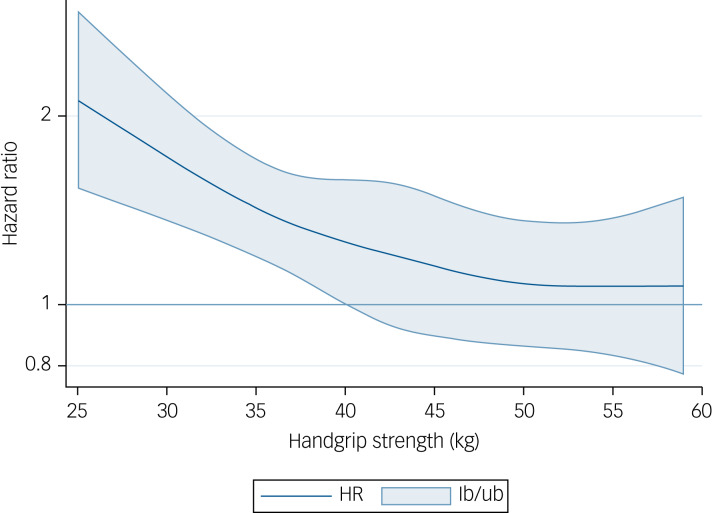
Dose–response association (adjusted hazard ratios and associated 95% confidence interval band) between handgrip strength (kg) and risk of depression in men aged 50 years or over. Adjusted for model B (age, education, country, body mass index, wave, physical inactivity, smoking, alcohol, partner, chronic diseases, prescribed drugs consumption and fruits and vegetables consumption). HR, hazard ratio; lb, lower boundary; ub, upper boundary.

**Fig. 4:**
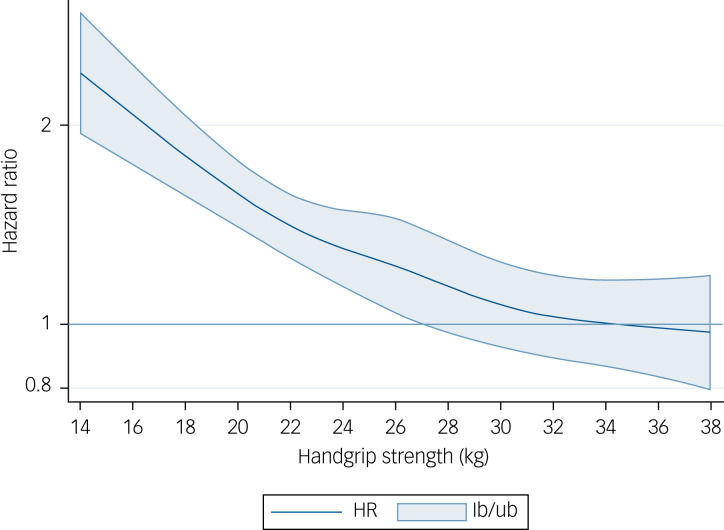
Dose–response association (adjusted hazard ratios and associated 95% confidence interval band) between handgrip strength (kg) and risk of depression in women aged 50 years or over. Adjusted for Model B (age, education, country, body mass index, wave, physical inactivity, smoking, alcohol, partner, chronic diseases, prescribed drugs consumption and fruits and vegetables consumption). HR, hazard ratio; lb, lower boundary; ub, upper boundary.

Analyses using restricted cubic spline modelling showed a significant association for each kg increase of handgrip strength and risk of depression up to 40 kg in men (HR 1.39, 95% CI 1.08–1.71) (Figure 3) and up to 27 kg in women (HR 1.28, 95% CI 1.05–1.55) (Figure 4), values from which no significant risk reduction for depression was observed.

### Sensitivity analyses

Results of sensitivity analyses using complete-case analyses did not substantially differ from those of the main analysis. Similarly, analyses removing participants with cases of depression within the first 2 years of follow-up or accounting for mortality and attrition as competing risk provided similar estimates. Additional analyses including the Nelson–Aalen cumulative hazard estimate for the survival time in the imputation model scarcely differed from those displayed in Figure 2.

## Discussion

### Main findings

In a large sample of older adults from 24 countries, higher levels of handgrip strength were associated with lower risk of depression. When categorised, handgrip strength exhibited an inverse association with risk of depression. Moreover, continuous handgrip strength also displayed an inverse association with risk of depression and an upper boundary for significant depression reduction risk among both men and women. To date, this is the first longitudinal study investigating the association between handgrip strength and risk of depression in older adults using repeated measurements in a large number of countries.

### Comparison with findings from other studies

In line with our findings, low muscle strength at baseline was associated with a higher 7-year incident depression among 5228 middle-aged and older Chinese participants.^19^ Likewise, a study among 17 713 ageing Americans found that every 5 kg of reduced handgrip strength was associated with a 6% higher depression risk.^10^ Interestingly, we observed an inverse curvilinear significant association for each kg increase of handgrip strength and depression up to 40 kg in men and up to 27 kg in women. This indicates that handgrip strength gains might be particularly beneficial when initial values are low, with a wider range of improvement for men.

Notably, prior research conducted with middle-aged and older European adults observed that handgrip strength may serve as a predictor of depression for specific age and gender subgroups experiencing musculoskeletal conditions.^32^ Furthermore, an identified reciprocal interaction between depression and frailty in older adults may indicate that a decline in neuromuscular function leads to a higher risk for depression.^33^ Our results support prior research observing substantial depression reduction with higher handgrip strength for both genders,^9^ although there is also evidence suggesting differences; for instance, a previous study among community dwelling adults from Ireland reported stronger associations between handgrip strength and depression in women than men.^21^

Finally, and supporting our findings, a study with 34 129 adults from six non-European countries found that those with weak handgrip strength had a higher prevalence of depression than those with stronger handgrip strength.^22^

### Interpretation of our findings

There may be several underlying explanations – biological as well as psychological – for the present findings. First, handgrip strength has been used as an overall indicator of health status, including sarcopenia.^34^ A meta-analysis by Chen et al showed that studies incorporating handgrip strength in the diagnosis of sarcopenia tended to show a stronger association between sarcopenia and depression.^11^ This is in line with a recent study reporting that depression was associated with sarcopenia mainly because of its association with reduced muscle strength.^12^ Neurotrophins such as brain-derived neurotrophic factor and neurotrophin-3 are produced by skeletal muscle among other tissues, and are associated with mood improvements.^13^

Another plausible mechanism linking handgrip strength with depression is low-grade inflammation, which is present in about a quarter of patients with depression, and over half of patients with this inflammatory condition have showed mildly elevated C-reactive protein levels.^35^ Interestingly, previous literature suggested that loss of skeletal muscle is associated with high levels of inflammatory markers such as interleukin-6 and C-reactive protein.^36^ Moreover, other lifestyle factors such as dietary habits may partly explain our results. For instance, low handgrip strength has been related to vitamin D deficiency,^37^ which has been associated with depression.^38^ In addition, other dietary habits such as intakes of antioxidants or proteins could also play a role.^13^

Third, being physically strong may lead to a sensation of psychological well-being. Besides physical decline, ageing *per se* also results in a reduction in cognitive abilities; being physically active across the lifespan also promotes structural and functional changes in the brain, benefiting cognitive functioning and reducing the risk of neurodegeneration.^39^ This can be important as ageing adults with cognitive impairment can also experience neuromuscular impairments such as reduced motor unit recruitment or motor neuron firing,^40^ which presumably will contribute to becoming weaker.

Notably, several psychosocial factors can influence depression, although these have been seldom investigated.^17^ For instance, different studies found that being stronger was associated with different factors that could influence depression such as good self-rated health,^41^ less psychological distress (i.e. stress and negative affect) and psychological well-being (i.e. optimism and self-esteem).^42^ Furthermore, those participating in exercise interventions with greater access to supportive social relationships have greater reductions in depression severity, compared with those with lower access.^43^ Future longitudinal studies with robust designs and large sample size are needed to understand how psychosocial factors mediate relationship between handgrip strength and depression.

### Strengths and limitations

The main strengths of the present study are the use of a large and representative sample from 24 countries and the use of an objective measure of handgrip strength. Moreover, we also accounted for time-varying handgrip strength and relevant time-varying covariate measurements in our modelling strategy, which reduces the possibility of obtaining biased estimates. Furthermore, we also took measures to minimise the chance of reverse causality by removing data for participants with depression onset that occurred within the first 2 years of follow-up.

On the other hand, interpretations of the present findings should consider several limitations. Importantly, because a substantial number of values was imputed for the main analyses, there is still a chance of biased estimates, although additional analyses accounting for participants with complete values solely showed similar results to those found in the main analyses, which reduces this possibility. Moreover, the plausibility of some degree of residual confounding concerning individual characteristics, life events and occupational hazards exists, although it is unlikely that those can importantly vary the results. Also, the participation rate at baseline was moderate (56%), which might increase the risk of selection bias. Nevertheless, such losses are compensated through refresher samples.^23^

In addition, the chance of some attrition bias affecting the accuracy of our estimations is plausible, but the average retention rate in SHARE (81%) importantly reduces such a possibility.^23^ As a result of this, we included a weight variable in the analyses in order to compensate for both non-response and attrition. Moreover, there is still a chance of a certain degree of residual confounding because of lack of controlling for psychological variables such as dysfunctional attitudes and negative emotionality that have been observed to influence the onset of depression.^44^ Finally, we only accounted for the first onset of depression as the outcome, but future studies might also consider variations of handgrip strength in relation to different depression recurrence.

### Implications

These results show higher levels of handgrip strength associated with lower risk of depression in older adults. However, such benefits may be limited up to specific handgrip strength values for both men and women. These findings warrant strength training programmes aimed at older adults to reduce depression risk. Healthcare practitioners may consider using the observed handgrip strength thresholds to screen for potential depression risk in older adults.

## Data Availability

This paper uses data from SHARE Waves 1, 2, 4, 5, 6, and 7 (DOIs: 10.6103/SHARE.w1.710, 10.6103/SHARE.w2.710, 10.6103/SHARE.w4.710, 10.6103/SHARE.w5.710, 10.6103/SHARE.w6.710, 10.6103/SHARE.w7.711, see Börsch-Supan et al (2013) for methodological details.^24^

## Supplementary material

For supplementary material accompanying this paper visit https://doi.org/10.1192/bjp.2022.178. click here to view supplementary material
