# Supplementary material for: Dose–response association of handgrip strength and risk of depression: a longitudinal study of 115 601 older adults from 24 countries
Source: Br J Psychiatry. 2023 Mar;222(3):135–42. doi: 10.1192/bjp.2022.178 (PMC9929711; doi:10.1192/bjp.2022.178)
Supplement: Supplementary file 1 [file S0007125022001787sup001.docx]

**Supplementary Online Content**

**Dose-response association of handgrip strength and risk of depression: a longitudinal study of 115 601 older adults from 24 countries**

**Supplementary Table 1.** Country weights for study participants stratified by sex.

**Figure 1.** Simplified DAG used for the selection of covariables for the association between handgrip strength and risk of depression.

**Figure 2.** Association of handgrip strength with risk of depression with complete case analyses.

**Figure 3.** Association of handgrip strength with risk of depression after removing participants who developed depression within the first two years of follow-up.

**Figure 4.** Association of handgrip strength with risk of depression accounting for mortality and attrition as competing risk.

**Figure 5.** Association of handgrip strength with risk of depression accounting for Nelson–Aalen cumulative hazard estimate to the survival time in the imputation model.

| **Table 1.** Country weights for study participants stratified by sex^a^ | | |
| --- | --- | --- |
|  | **Men** | **Women** |
| **Country** |  |  |
| Austria | 3,971,306 | 4,200,660 |
| Belgium | 5,140,743 | 5,337,874 |
| Bulgaria | 3,727,030 | 3,931,942 |
| Croatia | 2,035,607 | 2,234,538 |
| Czech Republic | 18,478,468 | 19,686,977 |
| Denmark | 2,684,213 | 2,735,229 |
| Estonia | 629,579 | 725,196 |
| Finland | 2,567,723 | 2,678,763 |
| France | 30,587,080 | 32,592,276 |
| Germany | 40,296,958 | 4,2172,474 |
| Greece | 5,438,858 | 5,548,456 |
| Hungary | 4,797,763 | 5,309,383 |
| Ireland | 2,031,416 | 2,038,846 |
| Israel | 3,418,244 | 3,511,856 |
| Italy | 28,144,391 | 29,825,093 |
| Luxembourg | 4,786,760 | 5,300,395 |
| Netherlands | 8,092,628 | 8,227,240 |
| Poland | 2,076,648 | 2,083,266 |
| Portugal | 5,077,470 | 5,425,560 |
| Romania | 10,391,006 | 10,928,679 |
| Slovenia | 978,181 | 1,022,293 |
| Spain | 21,535,302 | 22,117,853 |
| Switzerland | 3,640,621 | 3,796,494 |
| Sweden | 4,477,600 | 4,551,972 |

^a^Year 2004

**Figure 1.** Simplified DAG used for the selection of covariables for the association between handgrip and risk of depression


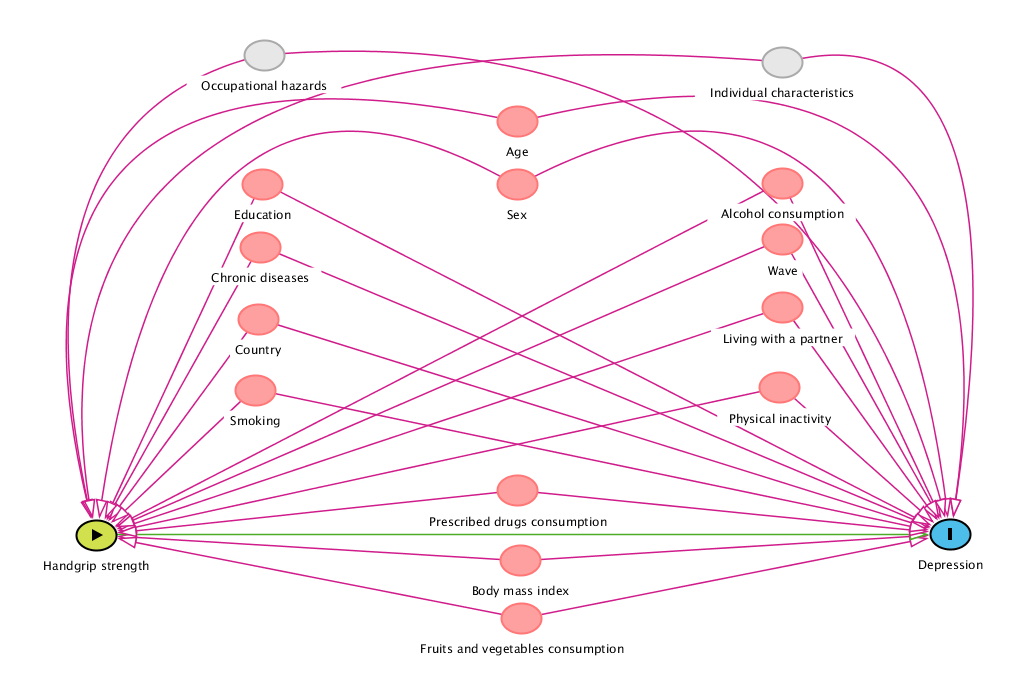


Nodes: Green (with ►) = exposure, Blue (with I) = outcome, Red = ancestor of exposure and outcome (confounder), White = adjusted variable, Grey = unobserved (latent) variable.

Arrows: Green = causal path, red = biasing path, black = blocked path


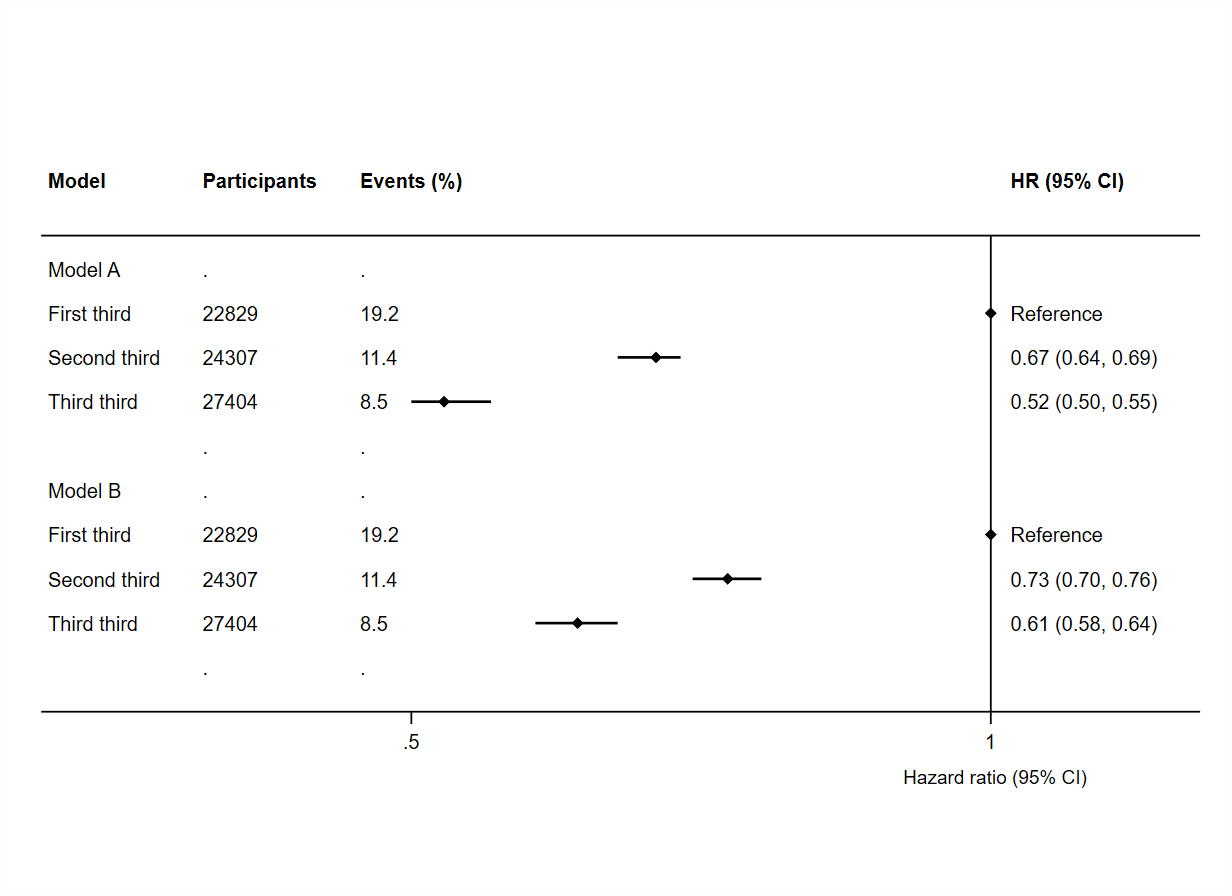
**Figure 2.** Association of handgrip strength with risk of depression with complete case analyses

Model A, adjusted for age and sex.

Model B, adjusted for age, sex, education, country, body mass index, wave, physical inactivity, smoking, alcohol, partner, chronic diseases, prescribed drugs consumption and fruits and vegetables consumption.

**Figure 3.** Association of handgrip strength with risk of depression after removing participants who developed depression within the first two years of follow-up


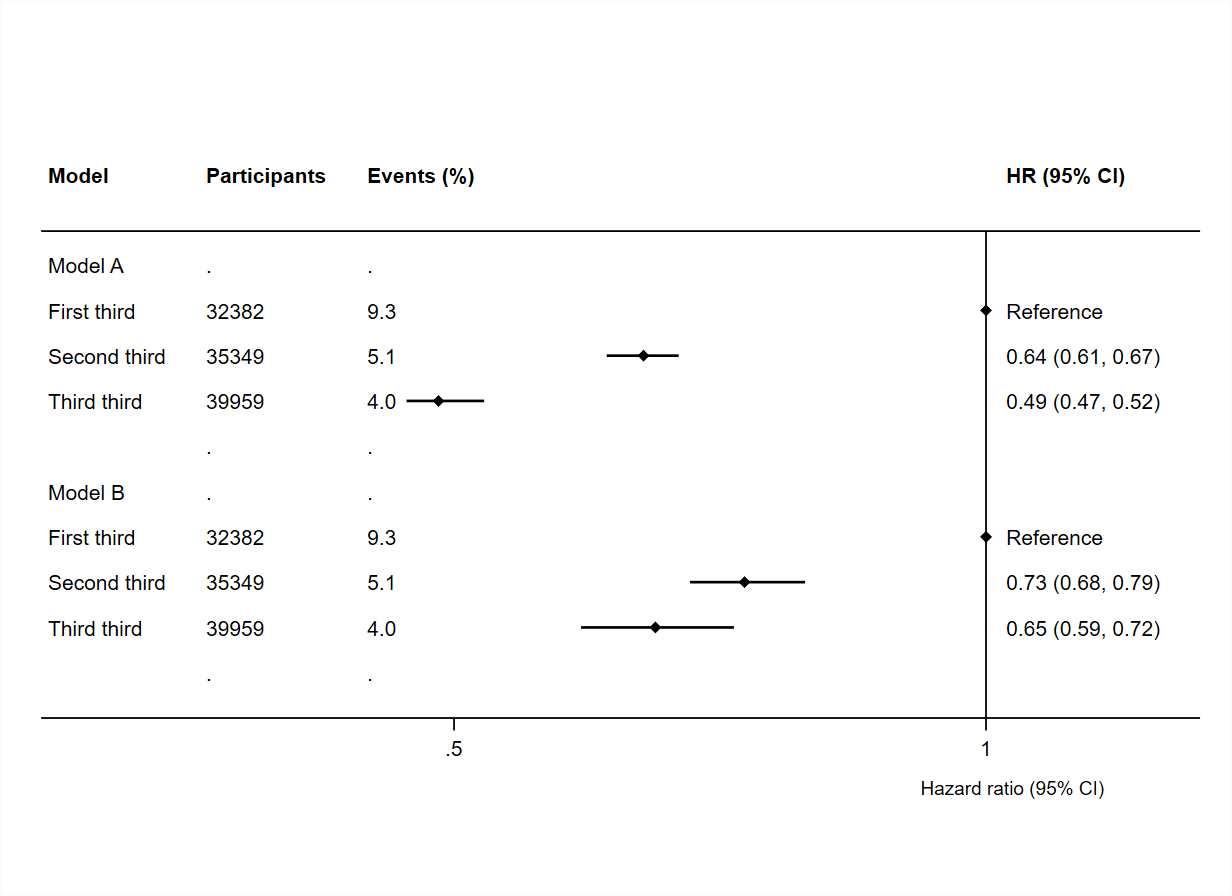


Model A, adjusted for age and sex.

Model B, adjusted for age, sex, education, country, body mass index, wave, physical inactivity, smoking, alcohol, partner, chronic diseases, prescribed drugs consumption and fruits and vegetables consumption.

**Figure 4.** Association of handgrip strength with risk of depression accounting for mortality and attrition as competing risk

**
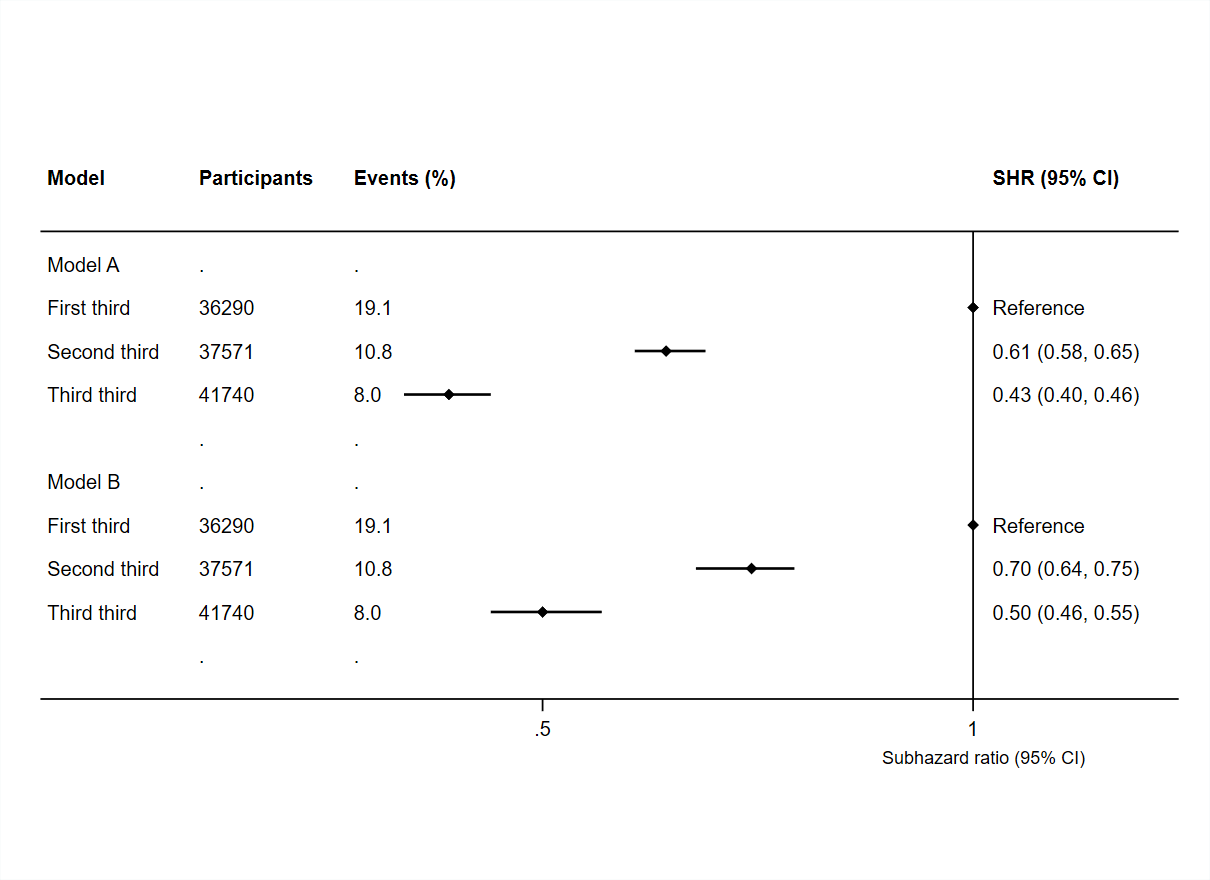
**

Model A, adjusted for age and sex.

Model B, adjusted for age, sex, education, country, body mass index, wave, physical inactivity, smoking, alcohol, partner, chronic diseases, prescribed drugs consumption and fruits and vegetables consumption.

**eFigure 5.** Association of handgrip strength with risk of depression accounting for Nelson–Aalen cumulative hazard estimate to the survival time in the imputation model


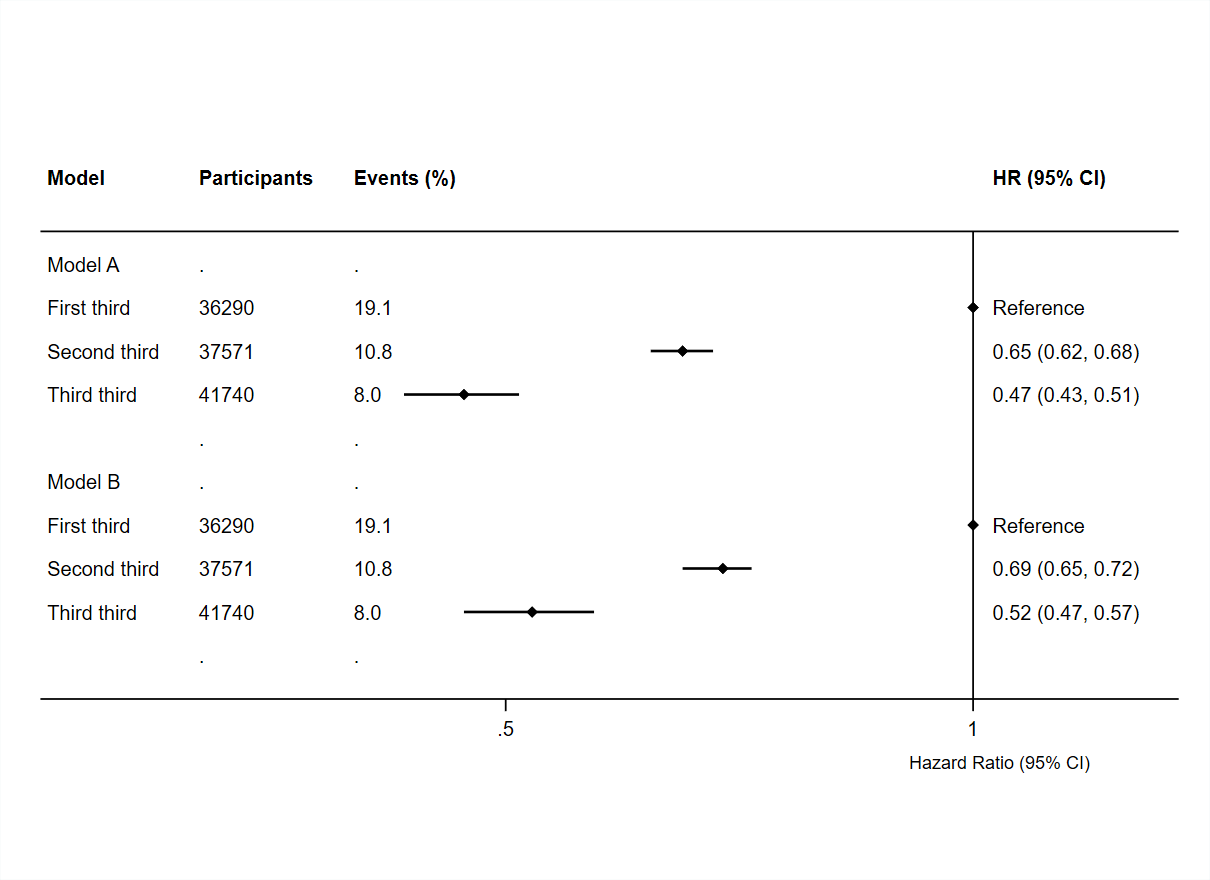


Model A, adjusted for age and sex.

Model B, adjusted for age, sex, education, country, body mass index, wave, physical inactivity, smoking, alcohol, partner, chronic diseases, prescribed drugs consumption and fruits and vegetables consumption.
